# Single- and Multi-Channel Modulation Detection in Cochlear Implant Users

**DOI:** 10.1371/journal.pone.0099338

**Published:** 2014-06-11

**Authors:** John J. Galvin, Sandy Oba, Qian-Jie Fu, Deniz Başkent

**Affiliations:** 1 Division of Communication and Auditory Neuroscience, House Research Institute, Los Angeles, California, United States of America; 2 Department of Head and Neck Surgery, David Geffen School of Medicine, University of California Los Angeles, Los Angeles, California, United States of America; 3 Department of Otorhinolaryngology, Head and Neck Surgery, University Medical Center Groningen, University of Groningen, Groningen, The Netherlands; 4 Research School of Behavioral and Cognitive Neurosciences, Graduate School of Medical Sciences, University of Groningen, Groningen, The Netherlands; University of Salamanca- Institute for Neuroscience of Castille and Leon and Medical School, Spain

## Abstract

Single-channel modulation detection thresholds (MDTs) have been shown to predict cochlear implant (CI) users’ speech performance. However, little is known about multi-channel modulation sensitivity. Two factors likely contribute to multichannel modulation sensitivity: multichannel loudness summation and the across-site variance in single-channel MDTs. In this study, single- and multi-channel MDTs were measured in 9 CI users at relatively low and high presentation levels and modulation frequencies. Single-channel MDTs were measured at widely spaced electrode locations, and these same channels were used for the multichannel stimuli. Multichannel MDTs were measured twice, with and without adjustment for multichannel loudness summation (i.e., at the same loudness as for the single-channel MDTs or louder). Results showed that the effect of presentation level and modulation frequency were similar for single- and multi-channel MDTs. Multichannel MDTs were significantly poorer than single-channel MDTs when the current levels of the multichannel stimuli were reduced to match the loudness of the single-channel stimuli. This suggests that, at equal loudness, single-channel measures may over-estimate CI users’ multichannel modulation sensitivity. At equal loudness, there was no significant correlation between the amount of multichannel loudness summation and the deficit in multichannel MDTs, relative to the average single-channel MDT. With no loudness compensation, multichannel MDTs were significantly better than the best single-channel MDT. The across-site variance in single-channel MDTs varied substantially across subjects. However, the across-site variance was not correlated with the multichannel advantage over the best single channel. This suggests that CI listeners combined envelope information across channels instead of attending to the best channel.

## Introduction

Temporal amplitude modulation (AM) detection is one of the few psychophysical measures that have been shown to predict speech perception by users of cochlear implants (CIs) [1]–[2] or auditory brainstem implants [3]. Various stimulation parameters have been shown to affect modulation detection thresholds (MDTs) measured on a single electrode, including current level, modulation frequency, and stimulation rate [2], [4]–[14]. In these single-channel modulation detection studies, MDTs generally improve as the current level is increased and as the modulation frequency is reduced. However, given that nearly all CIs are multichannel, it is crucial to characterize multichannel MDTs and their relation to the single-channel MDTs.

One factor that may affect multichannel temporal processing is loudness summation. Clinical CI speech processors are generally fitted with regard to loudness (i.e., between barely audible and the most comfortable levels), and adjustments are often necessary to accommodate multichannel loudness summation. As such, current levels on individual channels may be lower when presented in a multichannel context compared to those when measured in isolation. Because MDTs are level-dependent [4], [6], [8]–[10], [15], modulation sensitivity on individual channels may be poorer after adjusting for multichannel loudness summation. Another factor that may affect multichannel temporal processing is across-site variability in single-channel modulation sensitivity. Garadat et al. [16] showed significant variability in single-channel MDTs across stimulation sites within and across CI subjects. It is unclear how single-channel across-site variability may contribute to multichannel modulation sensitivity. These two factors – loudness summation and across-site variability – may combine in some way such that CI users may attend to the channels with the best modulation sensitivity, but at lower current levels after adjusting for summation. Alternatively, CI users may combine temporal information from all channels when detecting modulation with multiple channels.

While single-channel temporal processing has been extensively studied, there are relatively few studies regarding multichannel temporal processing. Geurts and Wouters [17] measured single- and multi-channel AM frequency detection in CI users. They found that AM frequency detection was improved with multichannel stimulation, relative to single-channel performance. However, no adjustment was made for multichannel loudness summation. Chatterjee and colleagues [15], [18] measured modulation detection interference (MDI) by fluctuating maskers in CI subjects. They found significant MDI, even when the maskers were spatially remote from the target, suggesting that CI users combined temporal information across distant neural populations (i.e., more central processing of temporal envelope information). Although their results supported the notion that central processes mediate envelope interactions, they did not find evidence for modulation tuning of the sort observed in normal-hearing (NH) listeners [19]–[20]. Kreft et al. [21] measured AM frequency discrimination in NH and CI listeners in the presence of steady-state and modulated maskers that were spatially proximate or remote to the target; the maskers were presented with or without a temporal offset relative to the target. Similar to the MDI findings by Chatterjee and colleagues, Kreft et al. [21] found significant interference by modulated maskers, but with some effect of masker location; temporal offset between the masker and target did not significantly reduce interference. The Chatterjee and Kreft studies present some evidence that central mechanisms result in combinations of and interactions between envelopes on remote spatial channels.

In this study, single- and multi-channel MDTs were measured in 9 CI subjects. MDTs were measured at relatively low and high presentation levels, and at low and high modulation frequencies. Single-channel MDTs were measured at 4 maximally spaced stimulation sites to target spatially remote neural populations, which would presumably result in greater across-site variability than with 4 closely spaced electrodes. Multichannel MDTs were measured using the same electrodes used to measure single-channel MDTs. To explore the effects of loudness summation on multichannel modulation sensitivity, multichannel MDTs were measured with and without adjustment for multichannel loudness summation.

## Methods

### Participants

Nine adult, post-lingually deafened CI users participated in this experiment. All were users of Cochlear Corp. devices and all had more than 2 years of experience with their implant device. Relevant subject details are shown in Table 1. All subjects previously participated in a related study [22].

**Table 1:** CI subject demographic information.

Subject	Gender	Age at testing (yrs) (yrs)	CI exp (yrs)	Dur deafness (yrs)	Device	Stim mode	Experimental electrodes			
							*A*	*B*	*C*	*D*
S1	F	77	10	12	N-24	MP1+2	8	12	17	22
S2	F	67	7	20	N-24	MP1+2	2	8	14	20
S3	M	81	15	1	N-22	BP+1	2	8	14	20
S4	F	78	23	14	Freedom	MP1+2	3	9	15	21
S5	M	70	21	4	N-22	BP+1	2	8	14	20
S6	F	58	17	20	N-22	BP+1	5	10	15	20
S7	F	28	5	5	Freedom	MP1+2	2	8	14	20
S8	F	66	7	24	Freedom	MP1+2	2	8	14	20
S9	M	74	3	2	Freedom	MP1+2	2	8	14	20

The experimental electrode used as the reference for loudness-balancing in shown in column C. CI exp = experience with cochlear implant device; Dur deafness = duration of diagnosed severe-to-profound deafness prior to cochlear implantation; Stim mode = stimulation mode; MP1+2 = intracochlear monopolar stimulation with two extracochlear grounds; BP+1 = intracochlear bipolar stimulation with active and return electrode separated by one electrode.

### Ethics Statement

All subjects provided written informed consent prior to participating in the study, in accordance with the guidelines of the St. Vincent Medical Center Institutional Review Board (Los Angeles, CA), which specifically approved this study. All subjects were financially compensated for their participation.

## Single-channel Modulation Detection Thresholds (MDTs)

### Stimuli

All stimuli were 300-ms biphasic pulse trains. The pulse phase duration was 100 µs; the inter-phase gap was 20 µs. Four test electrodes were selected and assigned to channel locations that spanned the electrode array from the base (A) to the basal-middle (B) to the middle-apical (C) to the apex (D). Table 1 lists the test electrode, channel assignment and stimulation mode for each subject. The stimulation rate was 500 pulses per second (pps). The presentation level was referenced to 25% or 50% of the dynamic range (DR) of a 500 pps stimulus. The modulation frequency was 10 Hz or 100 Hz.

Sinusoidal AM was applied as a percentage of the carrier pulse train amplitude according to [f(t)] [1+*m*sin(2πf_m_t)], where f(t) is a steady-state pulse train, *m* is the modulation index, and f_m_ is the modulation frequency. All stimuli were presented via research interface [23], bypassing CI subjects’ clinical speech processors and settings.

### Dynamic Range Estimation

DRs were estimated for all single-channel stimuli, presented without modulation (non-AM). Absolute detection thresholds were estimated according to the “counting” method commonly used for clinical fitting. Maximum acceptable loudness (MAL) levels, defined as the “loudest sound that could be tolerated for a short time,” were estimated by slowly increasing the current level until reaching MAL. Threshold and MAL levels were averaged across a minimum of two runs, and the DR was calculated as the difference in current (in microamps) between MAL and threshold.

### Loudness Balancing

The four test electrodes were loudness-balanced to a common reference using an adaptive two-alternative, forced-choice (2AFC), double-staircase procedure [24]–[25]. Stimuli were loudness-balanced without modulation. For each subject, the reference was the C channel (see Table 1) presented at 25% or 50% of its DR. The current amplitude of the probe was adjusted according to subject response (2-down/1-up or 1-down/2-up, depending on the track). The initial step size was 1.2 dB and the final step size was 0.4 dB. For each run, the final 8 of 12 reversals in current amplitude were averaged, and the mean of 2–6 runs was considered to be the loudness-balanced level. The low and high presentation levels were referenced to 25% DR or 50% DR of the reference electrode, and are referred to as the 25 loudness level (LL) and 50 LL, respectively. Thus, test electrodes A, B, C, and D were equally loud at the 25 LL and at the 50 LL presentation levels.

To protect against potential loudness cues in AM detection [14], [26], an adaptive AM loudness compensation procedure was used during the adaptive MDT task, as in Galvin et al. [22]. The AM loudness compensation functions were the same as in Galvin et al. [22], as the subjects, reference stimuli, and loudness-balance conditions were the same. Briefly, non-AM stimuli were loudness-balanced to AM stimuli using an adaptive, 2AFC double-staircase procedure [24]–[25]. The reference was the AM stimulus (AM depths = 5%, 10%, 20%, or 30%) presented to electrode C at either 25% or 50% DR. The probe was the non-AM stimulus, also presented to electrode C. The current amplitude of the probe was adjusted according to subject response (2-down/1-up or 1-down/2-up, depending on the track). For each run, the final 8 of 12 reversals in current amplitude were averaged, and the mean of 2–6 runs was considered to be the current level needed to loudness-balance the non-AM stimulus to the AM stimulus. For each loudness balance condition, an exponential function was fit across the non-AM loudness-balanced levels at each modulation depth. The mean exponent across the exponential fits was used to customize an AM loudness compensation function for each subject. For more details, please refer to Galvin et al. [22].

### Modulation Detection

MDTs were measured using an adaptive, 3AFC procedure. The modulation depth was adjusted according to subject response (3-down/1-up), converging on the threshold that corresponded to 79.4% correct [27]. One interval (randomly assigned) contained the AM stimulus and the other two intervals contained non-AM stimuli. Subjects were asked to indicate which interval was different. For each run, the final 8 of 12 reversals in AM depth were averaged to obtain the MDT; 3–6 test runs were conducted for each experimental condition.

MDTs were measured while controlling for potential AM loudness cues, as in Galvin et al. [22]. For each subject, the amount of level compensation *y* (in dB) was dynamically adjusted throughout the test run according to: 
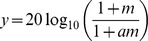
, where *m* is the modulation index of the modulated stimulus and α is the exponent (ranging from 0 to 1) of the exponential function fit to each subject’s AM vs. non-AM loudness-balance data. After applying this level compensation to the non-AM stimuli, the current level of all stimuli in each trial was independently roved by a random value between −0.75 and +0.75 dB (±4 clinical units) as in Fraser and McKay [14].

## Multichannel MDTs

### Stimuli

All stimuli were 300-ms biphasic pulse trains. The pulse phase duration was 100 µs; the inter-phase gap was 20 µs. The stimulation rate was 500 pps/electrode (ppse), resulting in a cumulative stimulation rate of 2000 pps. The modulation frequency was 10 Hz or 100 Hz. The component electrodes for the 4-channel stimuli were the same as used for single-channel modulation detection. The loudness-balanced current levels for each component electrodes were used for the 4-channel stimulus. The four channels were interleaved in time with an inter-pulse interval of 500 µs. Because of multichannel loudness summation, the 4-channel stimulus was louder than the single-channel stimuli [28]–[29]. To see the effects of loudness summation on modulation sensitivity, multichannel MDTs were also measured after loudness-balancing the 4-channel stimulus to the same single-channel references used for the single-channel loudness balancing. Thus, 4-channel MDTs were measured with and without adjustment for loudness summation.

Coherent sinusoidal AM was applied to all four electrodes as a percentage of the carrier pulse train amplitude according to [f(t)][1+*m*sin(2πf_m_t)], where f(t) is a steady-state pulse train, *m* is the modulation index, and f_m_ is the modulation frequency. All stimuli were presented via research interface [23].

### Loudness Balancing

The loudness-balanced current levels for the component electrodes were used as the initial stimulation levels for the 4-channel stimulus. The four-channel stimulus was loudness-balanced to the same single-channel reference stimuli used for single-channel loudness balancing (channel C, 500 pps, 25% or 50% DR) using the same adaptive procedure as for the single-channel loudness balancing. The current amplitude of the 4-channel probe was globally adjusted (in dB) according to subject response, thereby adjusting the amplitude for each electrode by the same ratio. Thus, the 4-channel stimulus was equally loud to the single-channel stimuli at the 25 LL and at the 50 LL presentation levels.

### Modulation Detection

Multichannel MDTs were measured using the same adaptive, 3AFC procedure as used for single-channel modulation detection. The modulation depth applied to all 4 electrodes was adjusted according to subject response. Potential AM loudness cues were controlled using the same AM loudness compensation and level roving methods used for single-channel modulation detection. Additionally, the reference current levels within the 4-channel stimulus were independently jittered by ±0.75 dB to reduce any loudness differences across the component electrodes.

## Results

**Figure 1:**
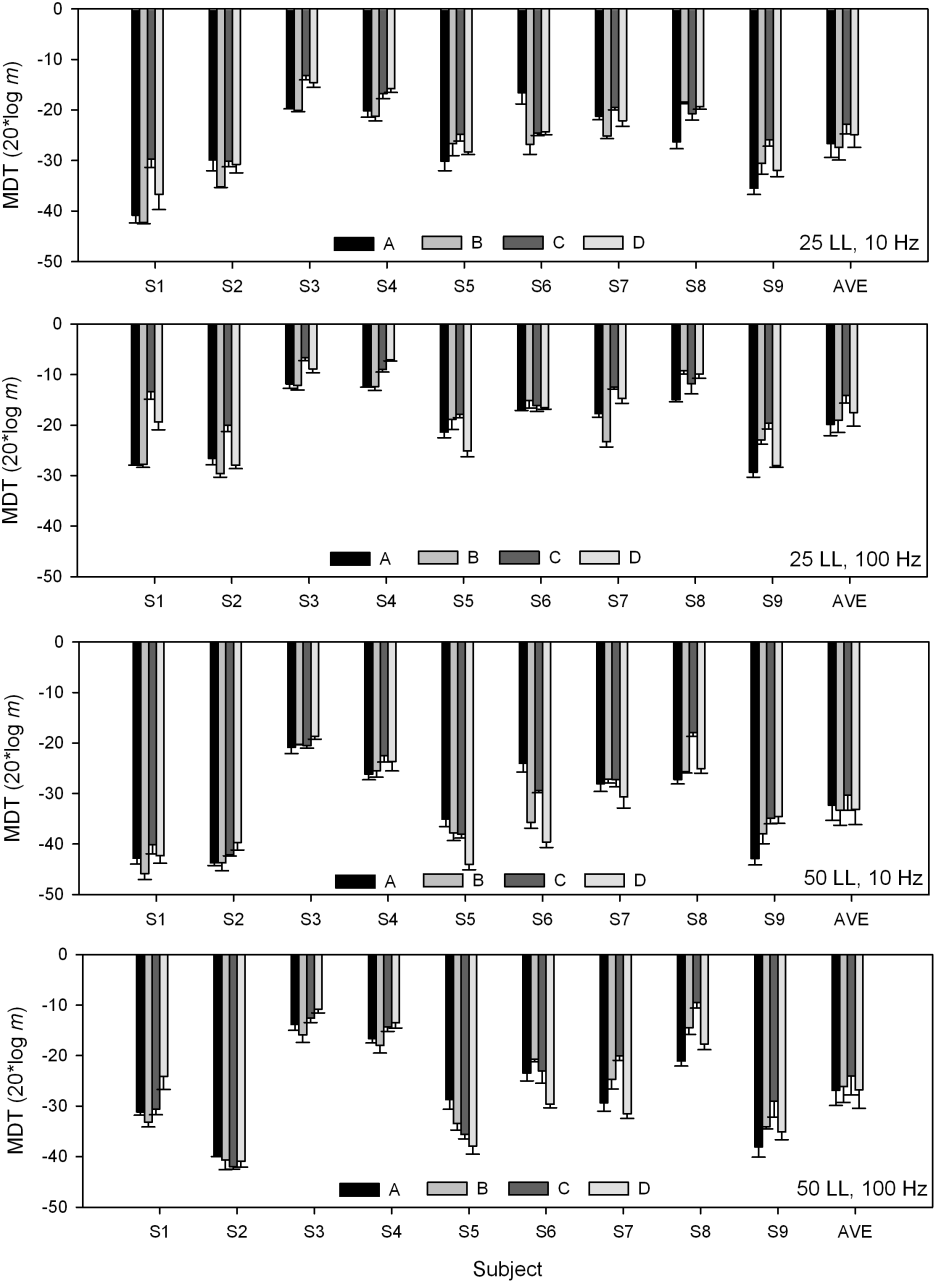
Single-channel MDTs for individual CI subjects. From top to bottom, the panels show 10-Hz MDTs at 25 LL, 100-Hz MDTs at 25 LL, 10-Hz MDTs at 50 LL, 100-Hz MDTs at 50 LL, respectively. The shaded bars show MDTs for the A, B, C, and D channels, respectively; the electrode-channel assignments are shown for each subject in Table 1. The error bars show the standard error.

Figure 1 shows individual and mean single-channel MDTs for the different listening conditions. Overall MDTs were highly variable across subjects, with subjects exhibiting relatively good (S1, S2, S5, S9) or poor modulation sensitivity (S3, S4, S8). Across modulation frequencies, mean MDTs were 7.57 dB better (lower) at the higher presentation level than at the lower level. Across presentation levels, mean MDTs were 7.05 dB better (lower) with the 10 Hz modulation frequency than with the 100 Hz modulation frequency. MDTs were variable across channel locations. Mean MDTs (across subjects) differed by as much as 5.74 dB across channels. For individual subjects, MDTs differed across channels by as little as 1.77 dB (S6, 25 LL, 100 Hz) to as much as 15.55 dB (S6, 50 LL, 10 Hz). A three-way repeated-measures analysis of variance (RM ANOVA) was performed on the data, with presentation level (25 LL, 50 LL), modulation frequency (10 Hz, 100 Hz), and stimulation site (A, B, C, or D) as factors. Results showed significant effects of presentation level [F(1,8) = 46.488, p<0.001], modulation frequency [F(1,8) = 39.665, p<0.001], and stimulation site [F(3,24) = 4.545, p = 0.012]. There was a significant interaction only between presentation level and modulation frequency [F(1,8) = 7.043, p = 0.029], most likely due to ceiling effects with the higher presentation level, especially for the 10 Hz modulation frequency. At very small modulation depths, the amplitude resolution may limit modulation sensitivity as the current level difference between the peak and valley of the modulation may be the same as or even less than each current level unit, which is approximately 0.2 dB.

Although the 3-way RM ANOVA showed a significant main effect of channel, there were individual differences in terms of the across-site variability in MDTs, with different best and worst channels for individual subjects. Additional 3-way ANOVAs were performed on individual subject data, with presentation level, modulation frequency and stimulation site as factors; the results are shown in Table 2. Significant effects were observed for presentation level in all 9 subjects, modulation frequency in 8 of 9 subjects, and stimulation site in 6 of 9 subjects. Post-hoc analyses showed that the best and worst stimulation sites differed among subjects.

**Table 2:** Results of three-way ANOVAs performed on individual subjects’ single-channel MDT data.

Subject	Stimulation level				Modulation frequency				Stimulation site			
	dF, res	F	p	Post-hoc p<0.05	dF, res	F	p	Post-hoc p<0.05	dF, res	F	p	Post-hoc p<0.05
S1	1, 3	65	*0.004*	*50 LL>25 LL*	1, 3	304	*<0.001*	*10 Hz>100 Hz*	3,3	25	*0.012*	*A,B>C*
S2	1, 3	134	*<0.001*	*50 LL>25 LL*	1, 3	10	0.052		3,3	2	0.29	
S3	1, 3	26	*0.015*	*50 LL>25 LL*	1, 3	113	*0.002*	*10 Hz>100 Hz*	3,3	10	*0.044*	
S4	1, 3	278	*<0.001*	*50 L>25 LL*	1, 3	634	*<0.001*	*10 Hz>100 Hz*	3,3	41	*0.006*	*A,B>C, D*
S5	1, 3	213	*<0.001*	*50 LL>25 LL*	1, 3	47	*0.006*	*10 Hz>100 Hz*	3,3	8	0.058	
S6	1, 3	220	*<0.001*	*50 L>25 LL*	1, 3	166	*<0.001*	*10 Hz>100 Hz*	3,3	27	*0.011*	*A>D*
S7	1, 3	54	*0.005*	*50 LL>25 LL*	1, 3	10	*0.049*	*10 Hz>100 Hz*	3,3	5	0.103	
S8	1, 3	22	*0.019*	*50 LL>25 LL*	1, 3	143	*0.001*	*10 Hz>100 Hz*	3,3	17	*0.021*	*A>C*
S9	1, 3	256	*<0.001*	*50 LL>25 LL*	1, 3	94	*0.002*	*10 Hz>100 Hz*	3,3	58	*0.004*	*A>B, A,D>C*

dF = degrees of freedom; res = residual error; F = F-ratio.

**Figure 2:**
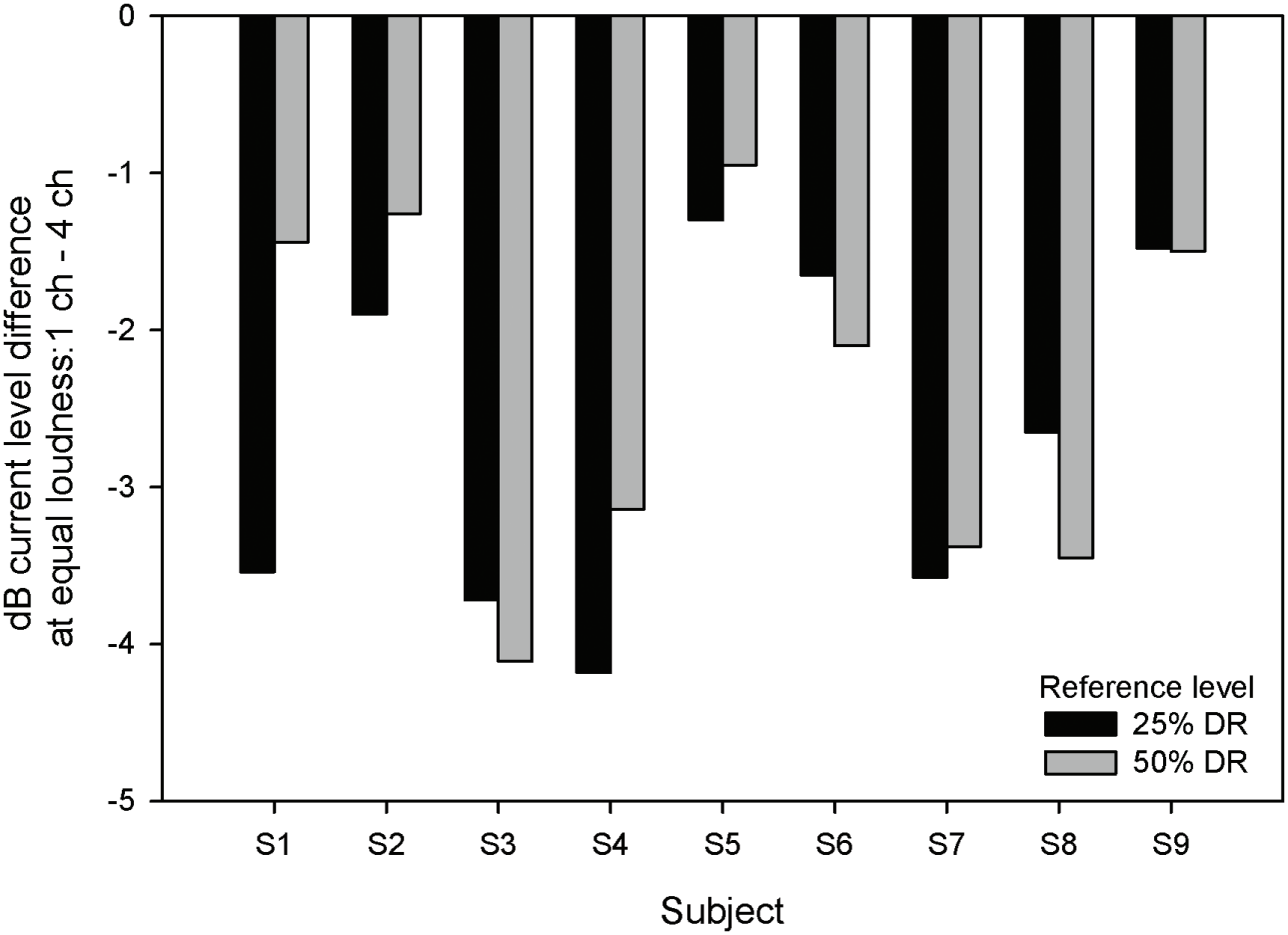
Loudness balancing between single- and multi-channel stimuli. The y-axis shows the current level adjustment needed to maintain equal loudness between 4-channel stimuli and the reference (single-channel, 500 pps, electrode C). The black bars show data referenced to 25% DR and the gray bars show data referenced to 50% DR. The error bars show the standard error.

Figure 2 shows the current level adjustment to the 4-channel stimulus needed to maintain equal loudness to the 500 pps, single-channel reference (electrode C at 25% and 50% DR). For the 4-channel stimuli, the current level adjustments were highly variable, ranging from 0.95 dB (subject S5 at the 50% DR reference) to 4.95 dB (subject S4 at the 25% DR reference). A one-way RM ANOVA showed no significant effect for reference level [F(1,8) = 2.398, p = 0.160], suggesting that loudness summation was similar at the relatively low and high presentation levels.

**Figure 3:**
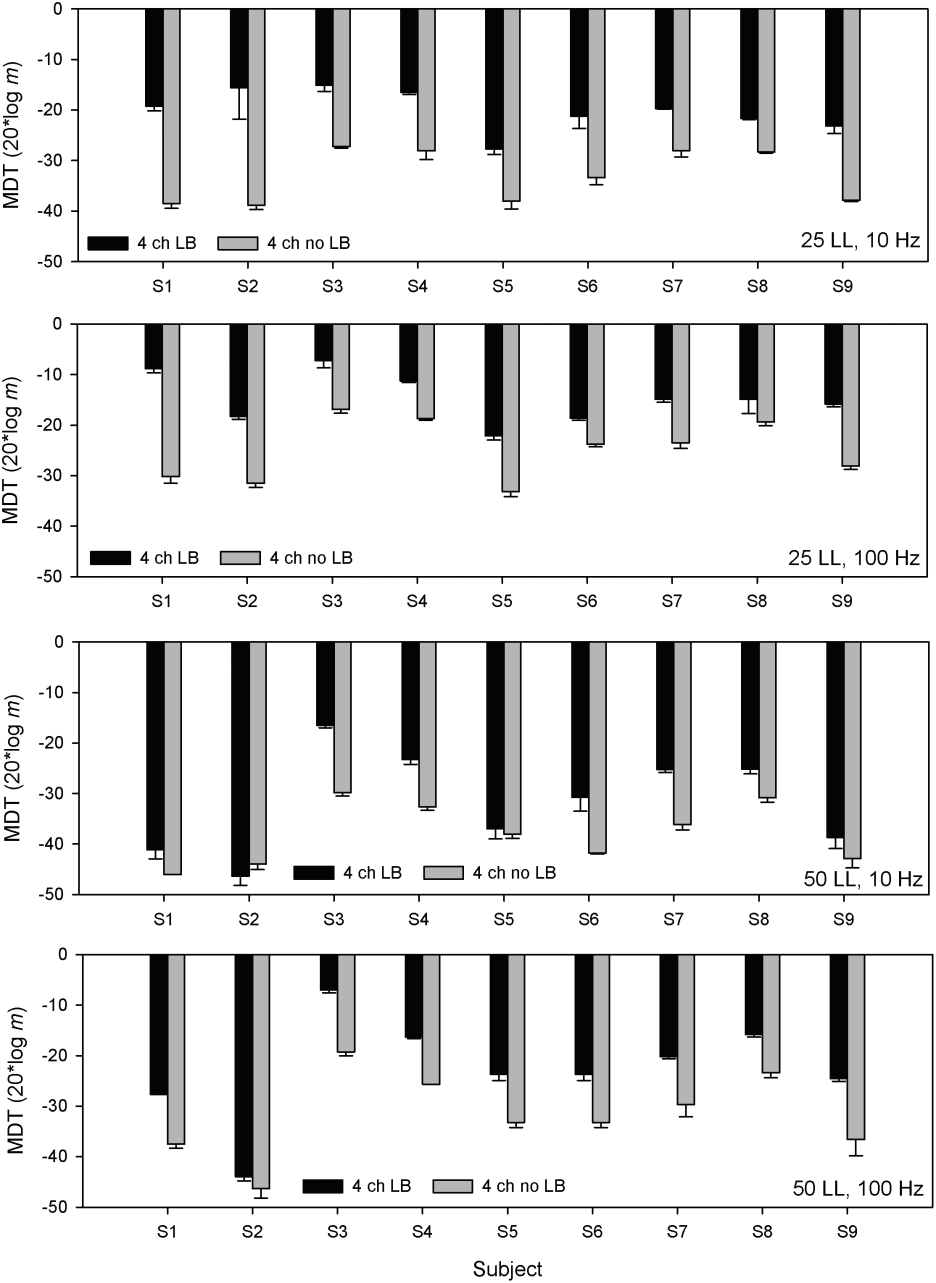
Multichannel MDTs for individual CI subjects. From top to bottom, the panels show 10-Hz MDTs at 25 LL, 100-Hz MDTs at 25 LL, 10-Hz MDTs at 50 LL, 100-Hz MDTs at 50 LL, respectively. The black bars show the MDTs for the 4-channel loudness-balanced stimuli (i.e., equally loud as the single-channel stimuli in Fig. 1) and the gray bars show MDTs for the 4-channel stimuli without loudness-balancing (i.e., louder than the single-channel stimuli in Fig. 1 and the 4-channel loudness-balanced stimuli). The error bars show the standard error.

Figure 3 shows individual subjects’ multichannel MDTs for the different listening conditions. The black bars show MDTs for the 4-channel loudness-balanced stimuli, which were as loud as the single-channel stimuli shown in Figure 1. The gray bars show MDTs for the 4-channel stimuli without loudness-balancing, which were louder than the single-channel stimuli shown in Figure 1 and the 4-channel loudness-balanced stimuli. As with the single-channel MDTs, multichannel MDTs were generally better with the higher presentation level (50 LL) and the lower modulation frequency (10 Hz). In every case, 4-channel MDTs were poorer when current levels were reduced to match the loudness of the single-channel stimuli. A three-way RM ANOVA was performed on the data, with presentation level (25 LL, 50 LL), modulation frequency (10 Hz, 100 Hz), and loudness summation (4-channel with or without loudness-balancing) as factors. Results showed significant effects of presentation level [F(1,8) = 18.13, p = 0.003], modulation frequency [F(1,8) = 54.967, p<0.001], and loudness summation [F(1,8) = 97.287, p<0.001].

**Figure 4:**
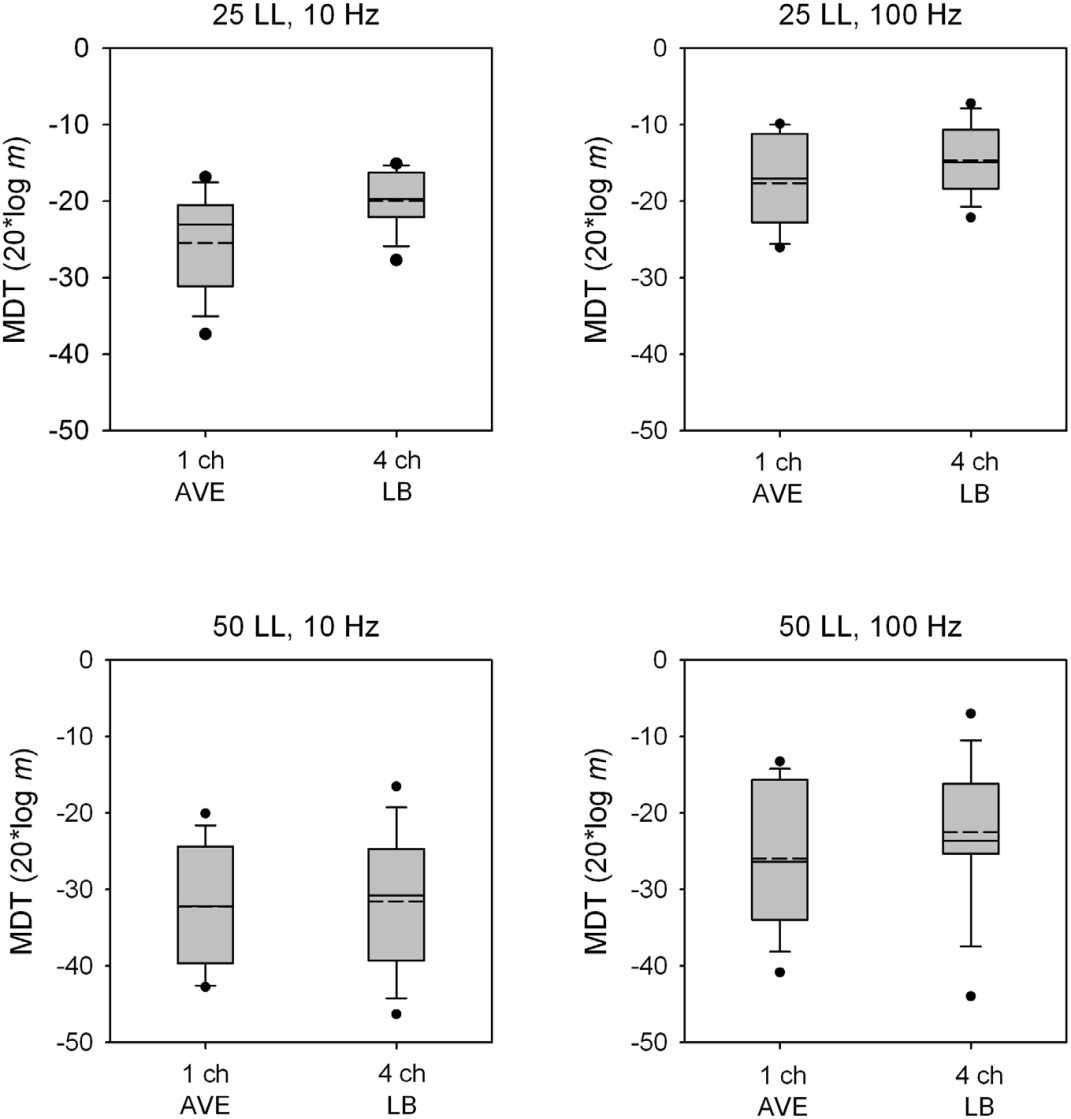
MDTs for equally loud single- and multi-channel stimuli. Box plots are shown for MDTs averaged across the best single channel or with the 4-channel loudness-balanced stimuli; note that all stimuli were equally loud. From left to right, the panels show data for the 25 LL/10 Hz, 25 LL/100 Hz, 50 LL/10 Hz, 50 LL/100 Hz conditions. In each box, the solid line shows the median, the dashed line shows the mean, the error bars show the 10^th^ and 90^th^ percentiles, and the black circles show outliers.

Figure 4 shows boxplots for MDTs averaged across single channels or with the 4-channel loudness-balanced stimuli. Note that all stimuli were equally loud. Across all conditions, the average single-channel MDT was 3.13 dB better (lower) than with the 4-channel loudness-balanced stimuli; mean differences ranged from 0.70 dB for the 50 LL/10 Hz condition to 5.44 dB for the 25 LL/10 Hz condition. A Wilcoxon signed rank test showed that the average single-channel MDT was significantly better than that with the 4-channel loudness-balanced stimuli (p = 0.003). Similarly, a ranked sign test showed that MDTs with the best single channel were significantly better than those with the 4-channel loudness-balanced stimuli (p<0.001). Finally, a ranked sign test showed that the difference between MDTs with the worst single channel and with the 4-channel loudness-balanced stimuli failed to achieve significance (p = 0.052).

**Figure 5:**
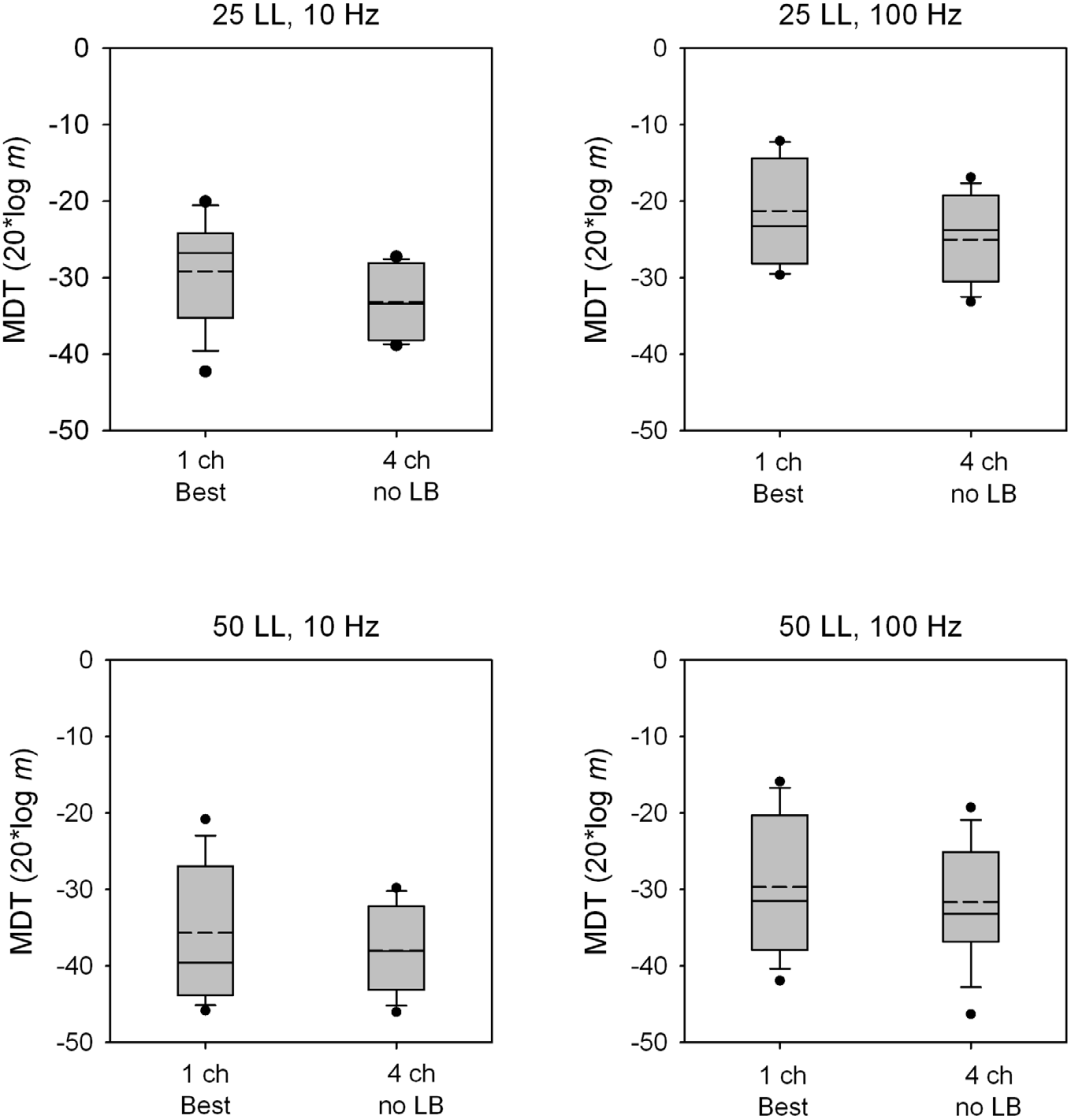
MDTs for single- and multi-channel stimuli without loudness summation compensation. Box plots are shown for MDTs with the best single-channel or with the 4-channel stimuli without loudness-balancing; note that the 4-channel stimuli without loudness-balancing were louder than the single-channel stimuli. From left to right, the panels show data for the 25 LL/10 Hz, 25 LL/100 Hz, 50 LL/10 Hz, 50 LL/100 Hz conditions. In each box, the solid line shows the median, the dashed line shows the mean, the error bars show the 10^th^ and 90^th^ percentiles, and the black circles show outliers.

Figure 5 shows boxplots for MDTs with the best single channel or with the 4-channel stimuli with no loudness compensation. Thus, the 4-channel stimuli were louder than the single-channel stimuli. Across all conditions, the mean MDT was 3.01 dB better with the 4-channel stimuli than with the best single channel; mean differences ranged from 1.97 dB for the 50 LL/100 Hz condition to 3.97 dB for the 25 LL/10 Hz condition. A paired t-test across all conditions showed that MDTs were significantly better with the 4-channel stimuli than with the best single channel (p = 0.001).

As shown in Figure 1, across-site variability in MDTs differed greatly across subjects. It is possible that subjects with greater across-site variability may attend more to the single channel with the best modulation sensitivity when listening to the 4-channel stimuli. Similarly, subjects with less across-site variability may better integrate information across all channels in the 4-channel stimuli. The mean across-site variance in single-channel MDTs was calculated for individual subjects across the presentation level and modulation frequency test conditions, as in Garadat et al. [16]. Across all subjects, the mean variance was 10.08 dB^2^, and ranged from 3.91 dB^2^ (subject S4) to 19.07 dB^2^ (subject S1). Individual subjects’ mean across-site variance was compared to the multichannel advantage (with no loudness compensation) in modulation detection over the best single channel without loudness-balancing (i.e., 4-channel MDT – best single-channel MDT). Linear regression analysis showed no significant relationship between the degree of multichannel advantage and across-site variance (r^2^ = 0.181, p = 0.253).

As shown in Figure 3, performance with 4-channel stimuli was much poorer when the current levels were reduced to match the loudness of single-channel stimuli. Figure 2 shows great inter-subject variability in terms of multichannel loudness summation. It is possible that the degree of multichannel loudness summation may be related to the deficit in multichannel modulation sensitivity after compensating for loudness summation. The mean loudness summation across both presentation levels was calculated for individual subjects, and was compared to the difference in MDTs between 4-channel stimuli with and without loudness-balancing. Linear regression analysis showed no significant correlation between the degree of multichannel loudness summation and the difference in MDTs between the 4-channel stimuli with or without loudness compensation (r^2^ = 0.014, p = 0.79).

## Discussion

The present data suggest that, at equal loudness, MDTs were poorer with 4 channels than with a single channel, most likely due to the lower current levels in the 4-channel stimuli needed to maintain equal loudness to the single-channel stimuli. With no compensation for loudness multichannel summation, MDTs were significantly better with 4-channel stimuli than with the best single channel, suggesting some multichannel advantage. Below, we discuss the results in greater detail.

### Effects of Presentation Level and Modulation Frequency

With single- or multi-channel stimulation, MDTs generally improved as the presentation level was increased and/or the modulation frequency was decreased, consistent with many previous studies [4], [6], [9]–[10], [12], [14]–[15], [22]. Across the single- and 4-channel conditions in Experiments 1 and 2, mean MDTs were 7.67 dB better with the 50 LL than with the 25 LL presentation level, and 7.07 dB better with the 10 Hz than with the 100 Hz modulation frequency.

### Effect of Loudness Summation on Multichannel MDTs

At equal loudness, 4-channel MDTs were significantly poorer than the average single-channel MDT (Fig. 4); 4-channel MDTs were also significantly poorer after compensating for multichannel loudness summation (Fig. 3). In both cases, the deficits were presumably due to lower current levels on each channel needed to compensate for multichannel loudness summation. MDTs are very level dependent, especially at lower presentation levels [6], [8]–[10], [15]. The present data suggest that at equal loudness, single-channel estimates of modulation sensitivity may greatly over-estimate the functional sensitivity when multiple channels are stimulated. In clinical speech processors, current levels must often be reduced to accommodate multichannel loudness summation. The present data suggests that such current level adjustments may worsen multichannel modulation sensitivity.

Loudness summation was not significantly correlated with the difference in MDTs between 4-channel stimuli with or without loudness compensation. This may reflect individual subject variability in modulation sensitivity, especially at presentation low levels. Such variability has been reported in many studies [6], [8]–[10], [13]–[14]. Thus, some subjects may have been more susceptible than others to the level differences between the 4-channel stimuli with and without loudness compensation.

Note that in the present study, we were unable to measure single-channel MDTs at the component channel stimulation levels used in the 4-channel loudness-balanced stimuli. After the current adjustment to accommodate multichannel loudness summation, the component channel current levels were often too low (i.e., below detection thresholds) to measure single-channel MDTs.

Multichannel loudness summation may also explain some of the advantage of multichannel stimulation observed by Geurts and Wouters [17] in AM frequency discrimination. Similar to their findings, the present data showed that multichannel stimulation without loudness compensation offered a small but significant advantage over the best single channel. In Guerts and Wouters [17] there was no level adjustment to equate loudness between the single- and multi-channel stimuli. If such a level adjustment had been applied to the multichannel stimuli, AM frequency discrimination may have better with single than with multiple channels, as in the present study with modulation detection. Future studies may wish to examine how component channels contribute to AM frequency discrimination in a multichannel context in which loudness summation does not play a role.

### Contribution of Single Channels to Multichannel MDTs

Across-site variability was not significantly correlated with the multichannel advantage over the best single channel, suggesting that CI subjects combined information across channels, instead of relying on the channels with best temporal processing, even when there was great variability in modulation sensitivity across stimulation sites. This finding is in agreement with recent multichannel MDI studies in CI users [18], [21] that suggest that multichannel envelope processing is more centrally than peripherally mediated.

### Implications for Cochlear Implant Signal Processing

The present data suggest that accommodating multichannel loudness summation, as is necessary when fitting clinical speech processors, may reduce CI users’ functional modulation sensitivity. When high stimulation rates are used on each channel, the functional temporal processing may be further compromised, as the current levels must be reduced to accommodate summation due to high per-channel rates and multichannel stimulation. Selecting a reduced set of optimal channels (ideally, those with the best temporal processing) to use within a clinical speech processor may reduce loudness summation, allowing for higher current levels to be used on each channel. Such optimal selection of channels has been studied by Garadat et al. [16], who found better speech understanding in noise when only the channels with better temporal processing were included in the speech processor. In that study, subjects were allowed to adjust the speech processor volume for the experimental maps, which may have compensated for the reduced loudness associated with the reduced-electrode maps, possibly resulting in higher stimulation levels on each channel. Bilateral signal processing may also allow for fewer numbers of electrodes within each side, thereby reducing loudness summation, increasing current levels, and thereby improving temporal processing. The reduced numbers of channels on each ear may be combined, as the spectral holes on one side are filled in by the other. Such optimized “zipper processors” have been explored by Zhou and Pfingst [30], who found better speech performance in some subjects, presumably due to the increased functional spectral resolution. Using fewer channels within each speech processor may have also reduced loudness summation, resulting in higher current levels and better temporal processing.

Loudness summation and spatio-temporal channel interactions should be carefully considered to improve the spectral resolution and temporal processing for future CI signal processing strategies. It is possible that by selecting a fewer number of optimal electrodes (in terms of temporal processing and key spectral cues) within each stimulation frame would reduce the instantaneous loudness summation, allowing for higher current levels that might produce better temporal processing. Using relatively low stimulation rates (e.g., 250–500 Hz/channel) might help reduce channel interaction between adjacent electrodes. Zigzag stimulation patterns which maximize the space between electrodes in sequential stimulation (e.g., electrode 1, then 9, then 5, then 13, then 3, then 11, etc.) might also help to channel interaction.

## Conclusions

Single- and multi-channel modulation detection was measured in CI users. Significant findings include:

1. Effects of presentation level and modulation frequency were similar for both single- and multi-channel MDTs; performance improved as the presentation level was increased or the modulation frequency was decreased.
2. At equal loudness, single-channel MDTs may greatly over-estimate multichannel modulation sensitivity, due to the lower current levels needed to accommodate loudness summation in the latter.
3. When there was no level compensation for loudness summation, multichannel MDTs were significantly better than MDTs with the best single channel.
4. There was great inter-subject variability in terms of multichannel loudness summation. However, the degree of loudness summation was not significantly correlated with the deficit in modulation sensitivity when current levels were reduced to accommodate multichannel loudness summation.
5. There was also great inter-subject variability in the across-site variance observed for single-channel MDTs. However, across-site variability was not significantly correlated with the multichannel advantage over the best single-channel. This suggests that CI listeners combined information across multiple channels rather that attend primarily to the channels with the best modulation sensitivity.

## References

[1] Cazals Y, Pelizzone M, Saudan O, Boex C (1994) Low-pass filtering in amplitude modulation detection associated with vowel and consonant identification in subjects with cochlear implants. J Acoust Soc Am 96: 2048–2054. 7963020 10.1121/1.410146

[2] Fu QJ (2002) Temporal processing and speech recognition in cochlear implant users Neuroreport. 13: 1635–1640. 10.1097/00001756-200209160-00013 12352617

[3] Colletti V, Shannon RV (2005) Open set speech perception with auditory brainstem implant. Laryngoscope 115: 1974–1978. 16319608 10.1097/01.mlg.0000178327.42926.ec

[4] Shannon RV (1992) Temporal modulation transfer functions in patients with cochlear implants. J Acoust Soc Am 91: 2156–2164. 1597606 10.1121/1.403807

[5] Busby PA, Tong Y, Clark GM (1993) The perception of temporal modulations by cochlear implant patients. J Acoust Soc Am 94: 124–131. 8354754 10.1121/1.408212

[6] Donaldson GS, Viemeister NF (2000) Intensity discrimination and detection of amplitude modulation in electric hearing. J Acoust Soc Am 108: 760–763. 10955643 10.1121/1.429609

[7] Chatterjee M, Robert ME (2001) Noise enhances modulation sensitivity in cochlear implant listeners: stochastic resonance in a prosthetic sensory system? J Assoc Res Otolaryngol 2: 159–171. 11550525 10.1007/s101620010079 PMC3201182

[8] Galvin JJ 3rd, Fu QJ (2005) Effects of stimulation rate mode and level on modulation detection by cochlear implant users. J Assoc Res Otolaryng 6: 269–279. 10.1007/s10162-005-0007-6 PMC2504596 16075190

[9] Galvin JJ 3rd, Fu QJ (2009) Influence of stimulation rate and loudness growth on modulation detection and intensity discrimination in cochlear implant users. Hear Res 250: 46–54. 19450432 10.1016/j.heares.2009.01.009 PMC5844469

[10] Pfingst BE, Xu L, Thompson CS (2007) Effects of carrier pulse rate and stimulation site on modulation detection by subjects with cochlear implants. J Acoust Soc Am 121: 2236–2246. 17471737 10.1121/1.2537501 PMC2562216

[11] Arora K, Vandali A, Dowell R, Dawson P (2011) Effects of stimulation rate on modulation detection and speech recognition by cochlear implant users. Int J Audiol 50: 123–132. 21070121 10.3109/14992027.2010.527860

[12] Chatterjee M, Oberzut C (2011) Detection and rate discrimination of amplitude modulation in electrical hearing. J Acoust Soc Am 130: 1567–1580. 21895095 10.1121/1.3621445 PMC3188971

[13] Green T, Faulkner A, Rosen S (2012) Variations in carrier pulse rate and the perception of amplitude modulation in cochlear implant users Ear Hear. 33: 221–230. 10.1097/AUD.0b013e318230fff8 22367093

[14] Fraser M, McKay CM (2012) Temporal modulation transfer functions in cochlear implantees using a method that limits overall loudness cues. Hear Res 283: 59–69. 22146425 10.1016/j.heares.2011.11.009 PMC3314947

[15] Chatterjee M, Oba SI (2005) Noise improves modulation detection by cochlear implant listeners at moderate carrier levels. J Acoust Soc Am 118: 993–1002. 16158655 10.1121/1.1929258

[16] Garadat SN, Zwolan TA, Pfingst BE (2012) Across-site patterns of modulation detection: Relation to speech recognition. J. Acoust. Soc. Am 131: 4030–4041. 10.1121/1.3701879 PMC3356319 22559376

[17] Geurts L, Wouters J (2001) Coding of the fundamental frequency in continuous interleaved sampling processors for cochlear implants. J Acoust Soc Am 109: 713–726. 11248975 10.1121/1.1340650

[18] Chatterjee M (2003) Modulation masking in cochlear implant listeners: envelope versus tonotopic components. J Acoust Soc Am 113: 2042–2053. 12703715 10.1121/1.1555613

[19] Dau T, Kollmeier B, Kohlrausch A (1997a) Modeling auditory processing of amplitude modulation. I. Detection and masking with narrow-band carriers. J Acoust Soc Am 102: 2892–2905. 9373976 10.1121/1.420344

[20] Dau T, Kollmeier B, Kohlrausch A (1997b) Modeling auditory processing of amplitude modulation. II. Spectral and temporal integration. J Acoust Soc Am 102: 2906–2919. 9373977 10.1121/1.420345

[21] Kreft HA, Nelson DA, Oxenham AJ (2013) Modulation frequency discrimination with modulated and unmodulated interference in normal hearing and in cochlear-implant users. J Assoc Res Otolaryngol 14: 591–601. 23632651 10.1007/s10162-013-0391-2 PMC3705089

[22] Galvin JJ 3rd, Fu QJ, Oba SI (2013) A method to dynamically control unwanted loudness cues when measuring amplitude modulation detection in cochlear implant users. J Neurosci Methods DOI information: 10.1016/j.jneumeth.2013.10.016. 10.1016/j.jneumeth.2013.10.016 PMC3897474 24269251

[23] Wygonski J, Robert ME (2002) HEI Nucleus Research Interface HEINRI Specification Internal materials.

[24] Jesteadt W (1980) An adaptive procedure for subjective judgments. Percept Psychophys 28: 85–88. 7413416 10.3758/bf03204321

[25] Zeng FG, Turner CW (1991) Binaural loudness matches in unilaterally impaired listeners Quarterly. J Exp Psych 43: 565–583. 10.1080/14640749108400987 1775657

[26] McKay CM, Henshall KR (2010) Amplitude modulation and loudness in cochlear implantees. J Assoc Res Otolaryng 11: 101–111. 10.1007/s10162-009-0188-5 PMC2820208 19798533

[27] Levitt H (1971) Transformed up-down methods in psychoacoustics. J Acoust Soc Am 49 Supp 2467. 5541744

[28] McKay CM, Remine MD, McDermott HJ (2001) Loudness summation for pulsatile electrical stimulation of the cochlea: effects of rate, electrode separation, level, and mode of stimulation. J Acoust Soc Am 110: 1514–1524. 11572362 10.1121/1.1394222

[29] McKay CM, Henshall KR, Farrell RJ, McDermott HJ (2003) A practical method of predicting the loudness of complex electrical stimuli. J Acoust Soc Am 113: 2054–2063. 12703716 10.1121/1.1558378

[30] Zhou N, Pfingst BE (2012) Psychophysically based site selection coupled with dichotic stimulation improves speech recognition in noise with bilateral cochlear implants. J Acoust Soc Am 132: 994–1008. 22894220 10.1121/1.4730907 PMC3427365

